# CAF-Associated Paracrine Signaling Worsens Outcome and Potentially Contributes to Chemoresistance in Epithelial Ovarian Cancer

**DOI:** 10.3389/fonc.2022.798680

**Published:** 2022-03-03

**Authors:** Michael Wessolly, Elena Mairinger, Sabrina Borchert, Agnes Bankfalvi, Pawel Mach, Kurt Werner Schmid, Rainer Kimmig, Paul Buderath, Fabian Dominik Mairinger

**Affiliations:** ^1^ Institute of Pathology, University Hospital Essen, Essen, Germany; ^2^ Department of Gynecology and Obstetrics, University Hospital Essen, Essen, Germany

**Keywords:** tumor microenvironment, epithelial ovarian cancer, high-grade serous ovarian cancer, cancer-associated fibroblasts, chemoresistance

## Abstract

**Background:**

High-grade serous ovarian cancer (HGSOC) is the predominant and deadliest form of ovarian cancer. Some of its histological subtypes can be distinguished by frequent occurrence of cancer-associated myofibroblasts (CAFs) and desmoplastic stroma reaction (DSR). In this study, we want to explore the relationship between therapy outcome and the activity of CAF-associated signaling pathways in a homogeneous HGSOC patient collective. Furthermore, we want to validate these findings in a general Epithelial ovarian cancer (EOC) cohort.

**Methods:**

The investigation cohort consists of 24 HGSOC patients. All of them were treated with platinum-based components and clinical follow-up was available. The validation cohort was comprised of 303 patients. Sequencing data (whole transcriptome) and clinical data were extracted from The Cancer Genome Atlas (TCGA). RNA of HGSOC patients was isolated using a Maxwell RSC instrument and the appropriate RNA isolation kit. For digital expression analysis a custom-designed gene panel was employed. All genes were linked to various DSR- and CAF- associated pathways. Expression analysis was performed on the NanoString nCounter platform. Finally, data were explored using the R programming environment (v. 4.0.3).

**Result:**

In total, 15 CAF-associated genes were associated with patients’ survival. More specifically, 6 genes (MMP13, CGA, EPHA3, PSMD9, PITX2, PHLPP1) were linked to poor therapy outcome. Though a variety of different pathways appeared to be associated with therapy failure, many were related to CAF paracrine signaling, including MAPK, Ras and TGF-β pathways. Similar results were obtained from the validation cohort.

**Discussion:**

In this study, we could successfully link CAF-associated pathways, as shown by increased Ras, MAPK and PI3K-Akt signaling to therapy failure (chemotherapy) in HGSOC and EOCs in general. As platinum-based chemotherapy has been the state-of-the-art therapy to treat HGSOC for decades, it is necessary to unveil the reasons behind resistance developments and poor outcome. In this work, CAF-associated signaling is shown to compromise therapy response. In the validation cohort, CAF-associated signaling is also associated with therapy failure in general EOC, possibly hinting towards a conserved mechanism. Therefore, it may be helpful to stratify HGSOC patients for CAF activity and consider alternative treatment options.

## 1 Introduction

According to the Global Cancer Statistics 2020 (GLOBOCAN), ovarian cancer ranks high as the deadliest tumor originating from gynecological sites, especially when comparing new cases (313.959) and disease-related deaths (207.252) (1). The staggering amount of patient deaths from this tumor type make it a serious health concern. Due to the lack of early symptoms, the disease is mostly discovered in advanced tumor stages with an extensive spread inside the peritoneal cavity (2). Epithelial ovarian cancer (EOC) is a heterogeneous disease, comprised of various subtypes. The four most prominent subtypes of EOC are clear cell, endometrioid, mucinous and serous ovarian cancer. The latter can be further subdivided into low-and high-grade serous ovarian cancer (3–5). In order to rate EOC subtypes regarding their proliferative and metastatic potential they can be differentiated as type I and type II EOC (6). Type 1 EOCs, encompassing endometrioid, clear cell and low-grade serous ovarian cancer, are characterized by slow progression and can often be discovered in early disease stages. Comparatively they have a better prognosis than type 2 EOCs (3, 5). High-grade serous ovarian cancer (HGSOC) is a type II EOC and the most prevalent EOC subtype, while also displaying a high proliferation rate and metastatic potential. Furthermore, HGSOC is mostly diagnosed at an advanced disease stage (3, 5). On a molecular level, DNA repair defects and p53 mutations are frequently encountered in HGSOC (4). Taken together, HGSOC is considered to have the poorest prognosis among the listed tumors and accounts for up to 80% of all deaths from EOCs (4, 7).

The two pillars of HGSOC therapy are cytoreductive surgery and adjuvant chemotherapy. The outcome is directly depended on disease stage (8). Since the 1980s platinum agents in combination with first cyclophosphamide and then paclitaxel are applied. The standard treatment consists of six cycles of carboplatinum and paclitaxel every three weeks (9, 10). In advanced stages, the anti-angiogenic agent bevacizumab may be added in addition to combined chemotherapy (10, 11). While most tumors regress initially after treatment, patients eventually face disease relapse, leading to the presumption that chemoresistance will develop eventually in the majority of cases (10, 12). From this point, patients are either defined as carrying a platinum-resistant or platinum-sensitive disease. Platinum-resistant patients present either with rapid progression after initial chemotherapy or a complete remission of the tumor mass, followed by a sudden relapse within six months after primary therapy has been completed. Similary, platinum-sensitive patients also display a complete remission after chemotherapy. However, disease relapse occurs later than in platinum-resistant patients (longer than six months after completing chemotherapy) (13–15).

In recurrent cases, platinum-based chemotherapy is also the treatment of choice for patients deemed platinum-sensitive. Moreover, platinum-sensitive tumors are especially vulnerable for treatment with Poly-ADP-Ribose-polymerase (PARP)-inhibitors in the first-line as well as the recurrent situation (16, 17). Platinum-resistant recurrent patients may also receive an alternative chemotherapeutic agent (cyclophophamide, doxorubicin, Pacitaxel or Topotecan) in combination with bevacizumab (18, 19).

One particular molecular subtype of HGSOC, the mesenchymal subtype is characterized by frequent generation of desmoplastic stroma. Mixed subtypes containing both epithelial and mesenchymal structures are also known. The occurrence of desmoplastic stroma in HGSOC is linked to decreased overall survival and resistance to platinum-based chemotherapy (20–22). This cancer-associated stroma is an important part of the tumor microenvironment. It may strongly influence tumor progression, invasion, metastasis, and angiogenesis (23, 24). A study by Zhang et al. (25) found increased expression of collagens (COL5A1, COL11A1), FAP, ACTA2 and p-SMAD2 within the stroma. FAP and ACTA2 (26, 27) are distinctive markers of a myofibroblast subtype, Cancer-associated fibroblasts (CAFs), which can reorganize the extracellular matrix to the tumors benefit or promote tumor-supportive inflammation (28, 29). Additionally, they secrete angiogenic factors (30). The constant reshuffling within the extracellular matrix triggers integrin-mediated activation of MAPK and PI3K-Akt signaling pathways, thereby enhancing cell proliferation and migration (31, 32).

Considering the abundance of CAFs in certain HGSOC subtypes and the link to dismal outcome, it seems very plausible that CAFs and associated stroma support tumor cells by paracrine signaling and providing a physical barrier, which facilitates the often-occurring platinum-resistance in HGSOC and decreased survival (33–35). We established a gene panel, encompassing various factors involved in prominent signaling pathways (TGF-β-, PI3K-Akt-, MAPK signaling) linked to desmoplastic stroma reaction (DSR). By analyzing the effects of paracrine CAF-signaling in a clinically well-defined and homogeneous collective of HGSOC patients, we intend to link it to impaired therapy outcome. Thereby, we also provide an mRNA-based expression signature, which may be helpful to stratify patients for application of platinum-based chemotherapy in the future.

## 2 Material and Methods

It should be noted that the following methods were applied as it was described in (36). However, in this study the gene panel is custom-designed in order to fit genes associated with DSR. This gene list derived from both previous research and current literature (37–40) includes key members of the canonical and non-canonical TGF-β signaling pathway, the PI3K pathway, WNT signaling, MAPK pathway, cell cycle progression, important growth factors and their respective receptors and well as main markers for activated myofibroblasts within the tumor (FAP, FN1, ACTA2/α-SMA).

### 2.1 Study and Cohort Design

#### 2.1.1 Investigation Cohort

**Figure 1:**
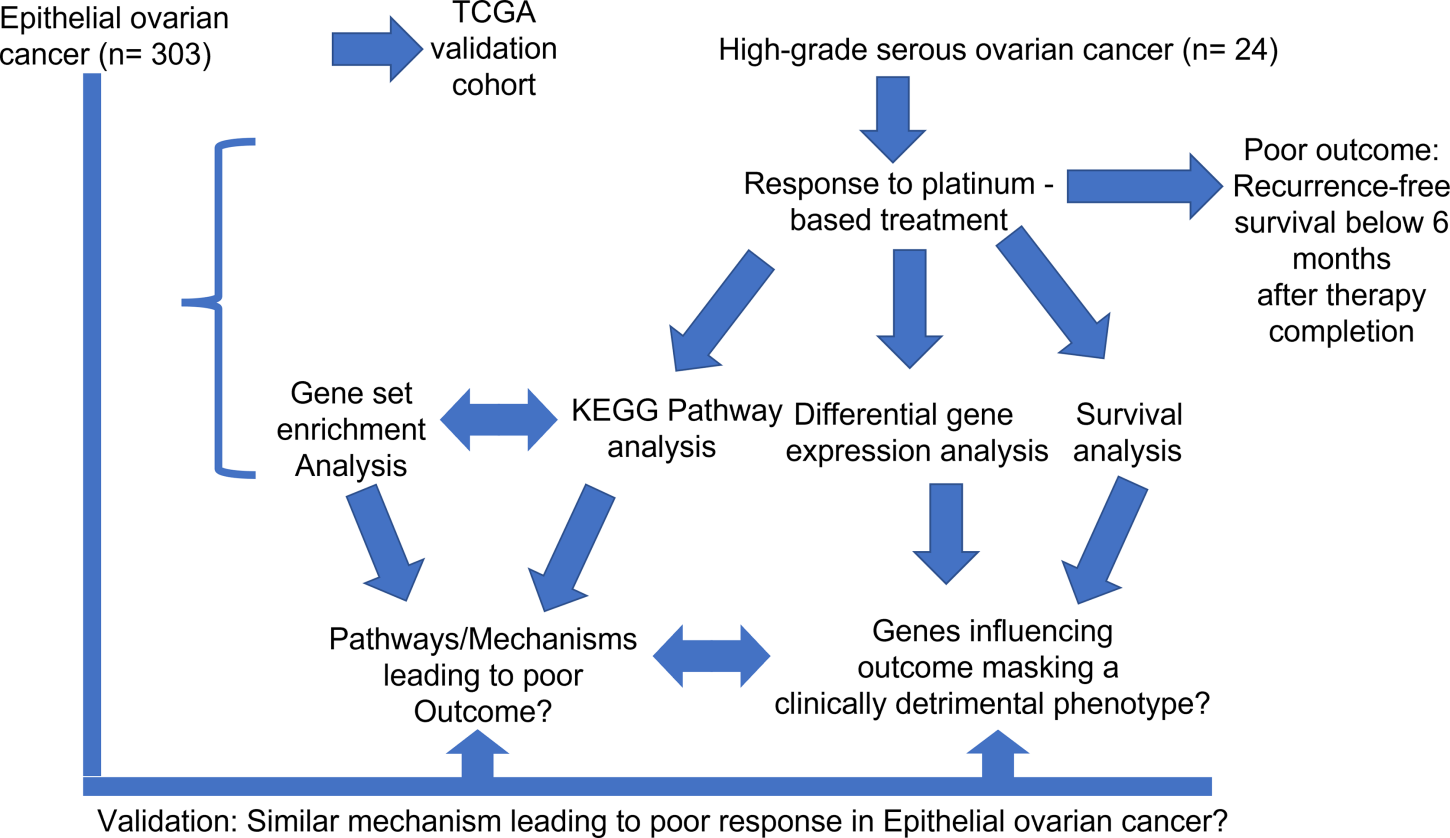
General methodical workflow of the study.

The retrospective investigation cohort encompassed 24 patients (**Figure 1**). They were diagnosed with high-grade serous ovarian cancer (HGSOC). The diagnosis was confirmed by an experienced pathologist according to the International Union Against Cancer (UICC), more specifically the 8^th^ edition of TNM guidelines (41). Patients were included into the study based on the following criteria: confirmed HGSOC with the ovaries as primary site, treatment with platinum-based chemotherapy (adjuvant) only, and sufficient follow-up data. Tumor tissue from the omentum was excluded. Clinical data include patients’ age, survival data (overall survival and recurrence-free survival) as well as tumor grading. DSR was identified in all 24 cases *via* staining of FAP, FN1 and ACTA2. Additionally, 7/24 patients had shown strong FAP positivity and extensive stroma remodeling. Based on recurrence-free survival (RFS), a binary outcome variable was defined that correlates to resistance against cisplatin. Patients displaying poor therapy outcome or therapy resistance were characterized by an RFS below 6 months after therapy completion, which conforms to the sources mentioned above (13–15). Median overall and recurrence-free survival for the investigation cohort were 35 and 9 months, respectively. All important clinical data from the investigation cohort are summarized in **Supplemental Table 1**.

#### 2.1.2 Validation Cohort

A cohort (n=303) of epithelial ovarian cancers (EOC) served as a validation cohort for this study. Gene expression data (RNA Seq) and clinical data were obtained from The Cancer Genome Atlas (TCGA) database (National Cancer Institute, National Human Genome Research institute, Bethesda, MD, US). The primary site of tumors within the validation cohort were the ovaries, though three tumor samples were derived from the omentum. A key selection criterion was sufficient follow-up that allowed for calculation of therapy outcome after platinum treatment. Poor therapy outcome or therapy resistance was defined as it was described in 2.1.1 (RFS below 6 months after therapy completion) (13–15). Median overall and recurrence-free survival for the validation cohort were 44 and 18 months, respectively. All important clinical data from the validation cohort are summarized in **Supplemental Table 2**.

### 2.2 RNA Extraction and Quantity Measurement

#### 2.2.1 Preparing Tissue Sections for RNA Isolation

Tumor tissue used for the study was formalin-fixed and paraffin-embedded (FFPE). All samples have been collected between 2005 and 2010. Only one tumor per patient was selected for further analysis. Each sample analyzed contained at least 85% tumor cells. All specimens have been stored at room temperature in the archives of the Institute of Pathology, University Hospital Essen. Tissue sections (thickness: 10 microns) were made using a “Microm HM340E” microtome (Thermo Fisher Scientific, Massachusetts, USA). The amount of sections was dependent on available tumor tissue (at least two sections per sample). The first tissue section from the surface layer has been discarded due to possible oxidation processes. In order to avoid loss of RNA yield, slides were stored by freezing (-20°C) until the RNA isolation procedure commenced.

#### 2.2.2 RNA Isolation

RNA was isolated in a semi-automatic workflow with the help of the Maxwell^®^ RSC Instrument (Promega, Wisconsin, USA) using a Maxwell^®^ RSC RNA FFPE kit (AS1440, Promega, Wisconsin, USA). The process was conducted according to the manufacturer’s instructions. In the final step, RNA was eluted in 50 µL RNase-free water.

#### 2.2.3 RNA Quantification

After the isolation process, RNA yield was quantified using a Qubit 2.0 fluorometer (Life Technologies, California, USA). Samples were prepared for Qubit measurement by utilizing an RNA broad-range assay kit (Invitrogen, Thermo Fisher Scientific, California, USA) according to the manufacturer’s instructions. In short, the fluorometric quantification is based on linear regression using predefined standards provided within the kit.

### 2.3 Digital Gene Expression Analysis

Samples harboring sufficient RNA yield were analyzed on the NanoString nCounter MAX/FLEX platform. 100 ng total RNA were used for each reaction. Digital expression analysis of 221 genes associated with DSR, TGF-β-, PI3K-Akt and MAPK signaling was performed utilizing a customized panel encompassing key genes of those pathways (**Supplemental Table 3**). Hybridization of capture- and reporter probes, carrying the biotin-tag and the 6-digits fluorescence barcode, respectively, with sample RNA was carried out using a thermocycler (Eppendorf, Germany) at 65°C (72°C lid temperature) for 21h as mentioned in the manufacturer’s protocol. After this stringent hybridization, post-hybridization processes including immobilization to the cartridge surface as well as clean-up of the hybridization products were conducted automatically on the NanoString nCounter Prep-Station according to the high sensitivity protocol. The cartridge was scanned directly after preparation on the NanoString nCounter Digital Analyzer with maximum sensitivity (555 fields of view).

### 2.4 NanoString Data Processing

Count data acquired by NanoString analysis were normalized and analyzed using the R statistical programming environment (The R Foundation for Statistical Computing, Institute for Statistics and Mathematics, Vienna, Austria; v. 4.0.3). Beside probes covering the target genes, a variety of technical and biological controls are included in the panel. First, eight different negative controls comprising probes with sequences not complimentary to the human transcriptome are included to estimate unspecific binding capability and identify potential alterations in the hybridization process. Second, six artificial RNA sequences with predefined concentration are included in the panel (technical positive controls). Those serve for detection of technical issues as well as to define the dynamic range of the assay and for calibration of linear regression, as those controls are diluted in a predefined manner and can be used as a standard curve. Samples without linear growth of those inherent positive controls indicating incomplete hybridization or elevated negative controls leading to decreased signal to noise ratio have been re-run. Third, nine reference genes for biological normalization purposes have been included in the panel, covering three high, three medium and three low expressed targets.

Technical normalization was performed by subtracting the mean counts from inherent negative controls plus two-times standard deviation from all target specific counts of each sample, while biological normalization was carried out using the geometric mean of included reference genes. In detail, a normalization factor has been calculated by dividing the geometric mean of all geometric means of the reference genes through the sample specific geometric mean of the reference genes. Afterwards, all target counts get multiplied by this normalization factor and afterwards mathematically rounded to integers. In addition to background subtraction, background noise was excluded by utilization of one-side Wilks *t*-test of negative controls and target specific counts in all samples to identify genes not relevantly expressed (p < 0.05) (36).

### 2.5 Statistical Analysis

Statistical and graphical analyses were also performed within the R statistical programming environment (v. 4.0.3)

First, the Shapiro–Wilks test was applied to test for normal distribution of data (42). For ordinal variables containing two groups, either the non-parametric Wilcoxon Mann-Whitney rank sum test or the parametric Student’s *t*-test was utilized (43). If ordinal variables contained more than two groups, the ANOVA (Analysis of variance, parametric) or the Kruskal–Wallis test (non-parametric) was used instead (44). Double dichotomous contingency tables were analyzed using Fisher’s exact test. To test dependency of ranked parameters with more than two groups the Pearson’s Chi-squared test was used. Group differences between metric variables were either detected by Pearson product moment correlation or Spearman’s rank correlation test (45). Quality control of run data was first performed by mean-vs-variance plotting to find outliers on target or sample level. True differences and clusters on both target and sample level were calculated by correlation matrices. To further specify the different candidate patterns, both unsupervised and supervised clustering, as well as principal component analysis were performed to overcome commonalities and differences. Sensitivity and specificity of markers were determined from receiver operating characteristic (ROC) curves illustrating their performance to discriminate the studied groups (46, 47). The bootstrap procedure (1000 iterations) was used for internal validation of the estimates in the ROC analyses. Pathway analysis is based on the KEGG database (Kyoto Encyclopaedia of Genes and Genomes) and was performed using the “pathview” package in R. Differences were specified by -log2 fold changes between means (parametric) or medians (non-parametric) of compared groups. Overall survival (OS) and RFS were calculated using single-factorial and combined fitting models. Survival analysis was done by Cox-regression (COXPH-model), and statistical significance was determined using likelihood ratio test, Wald test and Score (logrank) test. Kaplan-Meier curves and visualization *via* forest plots with a confidence interval of 95% (95% CI) were calculated based on existing survival data and combined survival curves. Beside p-value, hazard ratio (HR), time-dependent survival rate and median survival time have been calculated. Gene set enrichment analysis (GSEA) was performed using the WEB-based Gene SeT AnaLysis Toolkit (WebGestalt) website (48). In order to investigate certain signaling pathways, differential gene expression analysis was visualized on molecular network maps. These maps were provided by KEGG (49).

In order to overcome the problem of repeated statistical testing, p-values were corrected by utilizing the false discovery rate (FDR). Results were considered significant at p < 0.05 after adjustment (50).

## 3 Results

### 3.1 Gene Expression in CAF-Associated Pathways Negatively Impact Patient’s Overall Survival

Predictably, outcome after chemotherapy is linked to reduced overall survival (p < 0.05). However, in multivariate analysis, it turned out that this influence was independent of other clinical covariates like age at time of diagnosis, tumor grading or tumor stage (p < 0.05, **Supplemental Table 4**), thereby establishing therapy outcome as a sole determining factor influencing OS. Additionally, the influence of age, grading and stage on therapy outcome (RFS < 6 months after therapy completion) was also examined. None of those influenced therapy outcome in a multifactorial analysis, proving it an independent factor (**Supplemental Table 5**).

**Figure 2:**
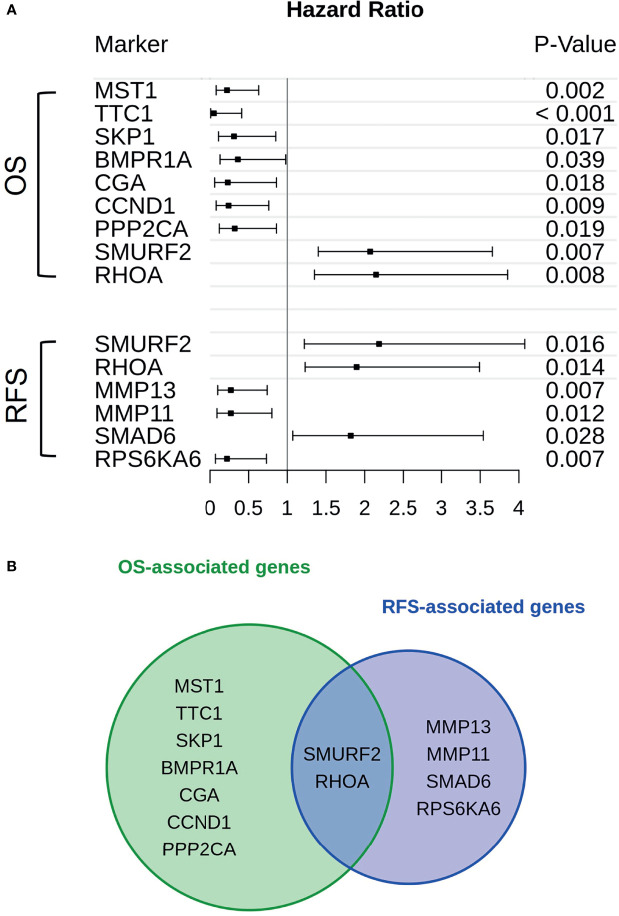
Genes in association with CAF-signaling impact patients’ survival. **(A)** For every gene that is hinted to impact patients’ overall survival (upper group) or recurrence-free survival (lower group) hazard ratios were calculated. The span of these values, including a risk estimate was visualized *via* forest plot. Of the original 24 patients available, only 19 were used in the calculations. Five patients were excluded due to missing survival data. The p-value was calculated by Score-logrank test. **(B)** Both groups of genes, either in association with overall survival (green) or recurrence-free survival (blue), were compared and overlaps between them were also highlighted.

Genes associated with TGF-β or PI3K-Akt signaling were subjected to a cox proportional hazard model in order to assert their expressions’ influence on either OS or RFS (**Figure 2A**). Overall, 13 genes were linked to reduced OS (n = 9) or RFS (n = 6). Of those genes only SMURF2 and RHOA overlapped between both survival variables (**Figure 2B**).

### 3.2 High Expression of CAF-Associated Genes Drives Therapy Failure, While Also Impacting Patients’ Survival

**Figure 3:**
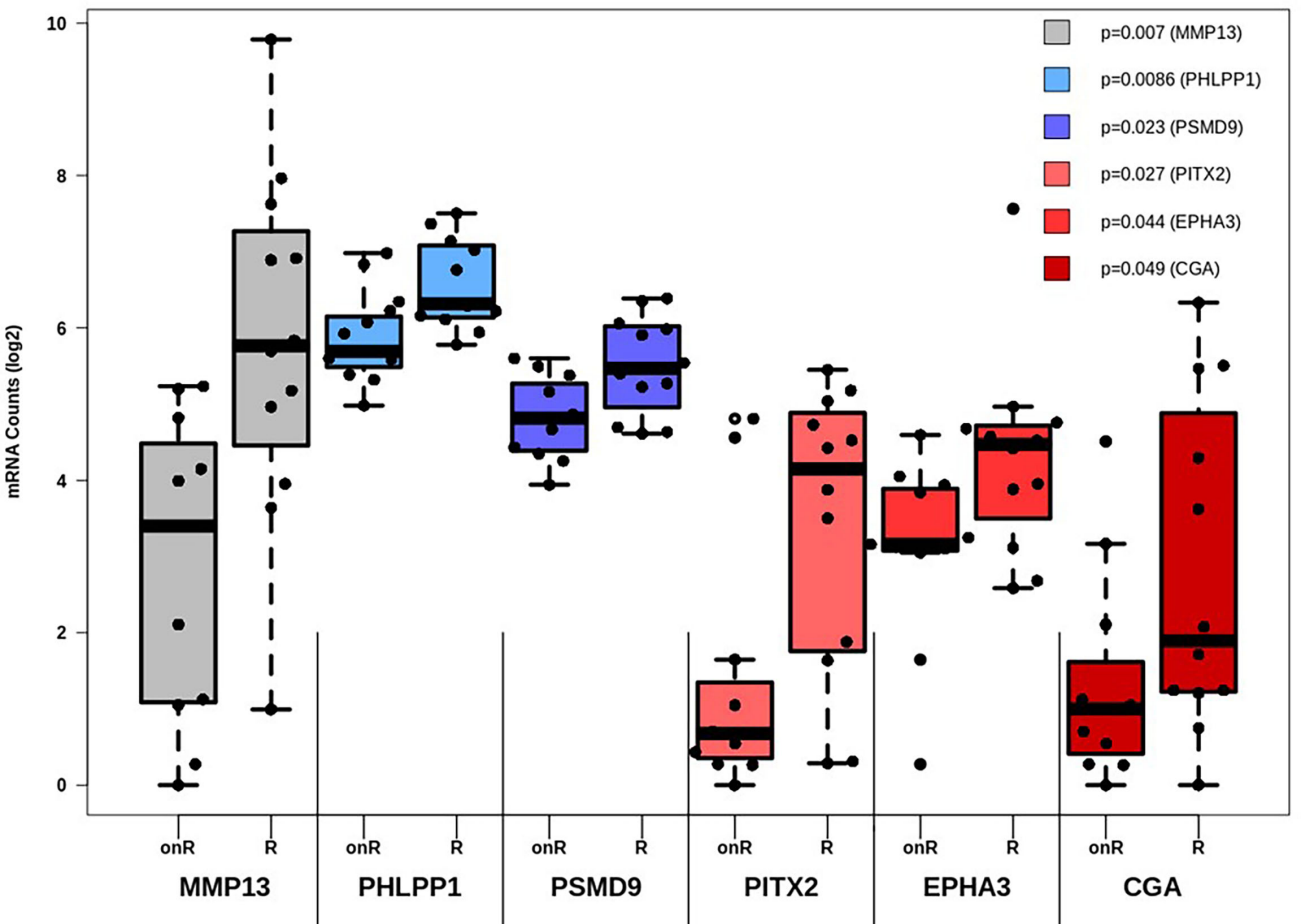
Differential expression analysis of genes affecting therapy outcome. For each gene the number of measured counts were compared between patients still responding to therapy (Ongoing Response, “onR”) or not (Resistant, “R”). Group-based expression differences were visualized by p-value, which was calculated by Wilcoxon Mann-Whitney rank sum test.

While the expression of certain genes influences patients’ survival, it may also be possible that specific genes may constitute a gene expression signature, which can be correlated to therapy outcome. As such, all genes involved in TGF-β and PI3K-Akt signaling were subjected to differential expression analysis in dependance of this outcome. All in all, six genes are linked to therapy failure (p < 0.05, **Table 1**). Furthermore, differential expression of those genes was analyzed whether they were not (Resistant, “R”) or still responding to chemotherapy (ongoing response, “onR”). Strikingly, the expression of all genes was increased in patients without long-term response to chemotherapy (**Figure 3**).

**Table 1:** Genes associated with poor therapy outcome after chemotherapy (p < 0.05).

Associated genes:	P-value:
MMP13	0.007
EPHA3	0.044
PSMD9	0.023
PITX2	0.027
PHLIPP1	0.0086
CGA	0.049

Two genes were also linked to OS (CGA, p= 0.018) and RFS (MMP13, p= 0.0074). Group-based survival differences were asserted by cox proportional hazard models. The patient groups were separated based on whether genes displayed high or low expression rates. In either case, high expression of both MMP13 and CGA were detrimental to patients’ survival (**Supplemental Figure 1**). Moreover, we validated the correlation between gene expression levels and the occurrence of desmoplastic tumor stroma. The presence of CAFs within the tumor, quantitatively depicted as FAP positivity, was strongly linked to the expression of MMP13, AKT1, TGFB3 and TGFBR2, among other factors (**Supplemental Table 6**).

### 3.3 Increased Activity of Signaling Pathways Involved in Growth Factor and Fibroblast Signaling Is Associated With Novel Cell Death Pathways and Cytokine-Cytokine Receptor Interactions

**Figure 4:**
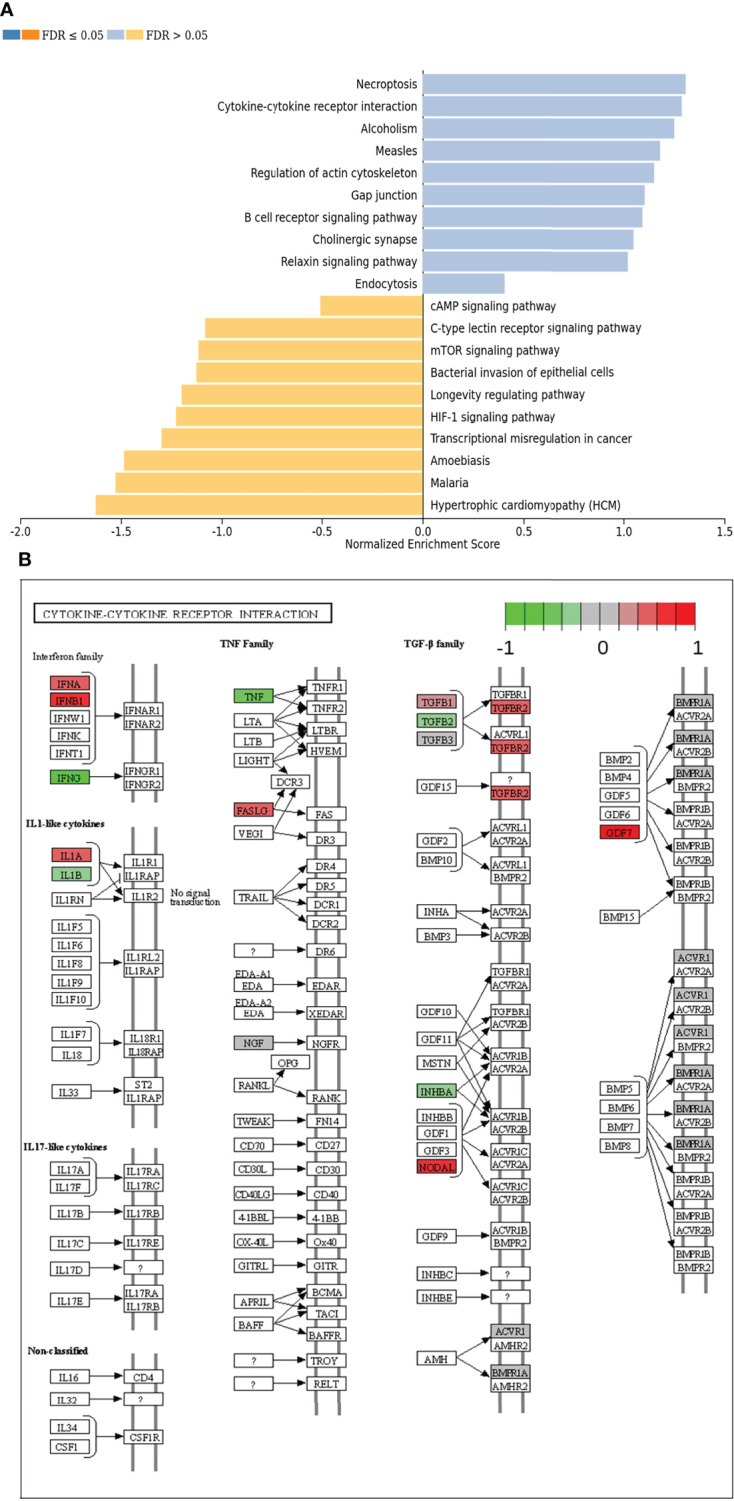
**(A)** Gene set enrichment analysis of differentially expressed genes regarding therapy outcome in various signaling pathways. Blue: Genes in association with therapy outcome are strongly expressed in those pathways. Yellow: Genes in association with therapy outcome are barely expressed in those pathways. FDR: False Discovery rate. Due to testing the expression of certain genes in specific pathways multiple times, the p-values are adjusted for the naturally occurring variance by the FDR method. **(B)** Genes expressed in association with “Cytokine-cytokine receptor interaction” and therapy failure in HGSOC. The color code indicates at differential gene expression whether the patients did not (red) or did respond well to chemotherapy (green). This molecular network map stems from the Kyoto Encyclopedia of Genes and Genomes (KEGG) database.

**Figure 5:**
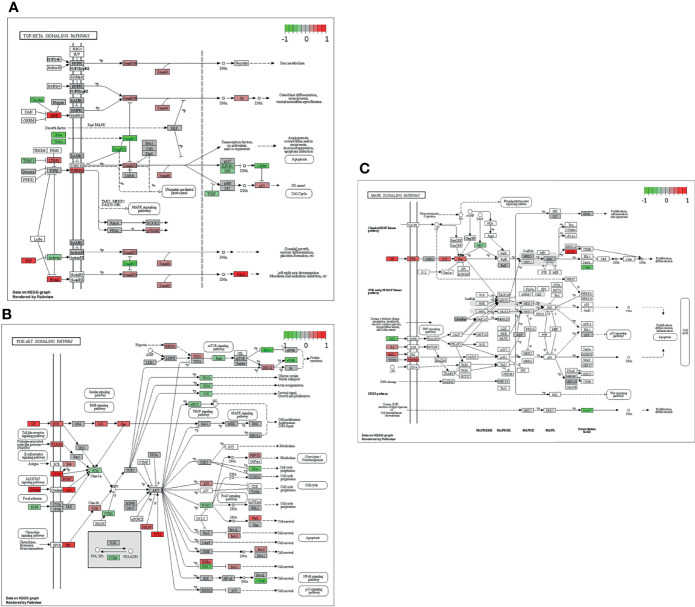
Genes expressed in association with the TGF-β **(A)**, PI3K-Akt **(B)** and MAPK **(C)** signaling pathways and therapy outcome in HGSOC. The color code indicates at differential gene expression whether the patients did not (red) or did respond well to chemotherapy (green). This molecular network map stems from the Kyoto Encyclopedia of Genes and Genomes (KEGG) database.

In the next step, single gene associations with outcome needed to be put into context with larger signaling pathways. For this purpose, a gene set enrichment analysis (GSEA) was performed (**Figure 4A**), followed by KEGG pathways analysis (**Figures 4B**, **5**). The latter allowed for accurate examination of differential expression, depending on durable therapy responses, in specific pathways.

According to GSEA, genes in association with worsen therapy outcome were highly expressed in signaling pathways linked to “Necroptosis”, “Cytokine-cytokine receptor interaction” and “Alcoholism”. However, they were barely expressed in signaling pathways linked to “Hypertrophic cardiomyopathy”, “Malaria” and “Amoebiasis” (**Figure 4A**).

It is especially interesting that one of the top listed pathways regarding overexpression of genes correlated to poor outcome is “Cytokine-cytokine receptor interaction” (**Figure 4A**). This necessitated a more precise look into the underlaying pathways (**Figure 4B**). Apparently, ligands for alpha-and beta interferon receptors are highly expressed. Furthermore, TGFB1 and TGFBR2 were highly expressed as well, which indicates high TGF-β activity. Other factors, which also showed high expression were FASLG, IL-1A, CXCL-12, NODAL and GDF7. TGF-β signaling may also hint towards fibroblast activity which is underlined by looking at “Pathways in Cancer” (**Supplemental Figure 2**). Two important factors, often linked to fibroblast activity FGF and PDGF (and PDGFR) display strong expression in association with poor therapy outcome. Their downstream signaling *via* Ras finally leads to activation of matrix-metalloproteinases (MMPs) like MMP13. Other important signaling pathways, which are also linked to fibroblast activity are the TGF-β- (**Figure 5A**), PI3K-Akt- (**Figure 5B**), and MAPK (**Figure 5C**) signaling pathways. Most genes within those pathways display strong expression in case of therapy failure. It should be noted that TGF-β signaling is still partially carried out by canonical SMAD signaling, with SMAD2/SMAD3 still being active, while the inhibitory SMAD6/7 are seemingly not expressed in the group responding poorly to chemotherapy (**Figure 5A**). However, in comparison, genes linked to non-canonical TGF-β signaling (MAPK and PI3K-Akt signaling) are more strongly expressed as indicated by intensive red coloring (**Figures 5B, C**)

### 3.4 Pronounced Non-Canonical TGF-ẞ Signaling Can Be Found in Patients Responding Poorly to Chemotherapy Across Various Epithelial Ovarian Cancer Subtypes

Similar observations regarding those specific pathways have been made in the validation cohort. In contrast to the actual investigation cohort, the validation cohort was heterogenous (see *Study and Cohort Design*). Thus, it was composed of different malignant entities, including HGSOC. Similarly, TGF-β, MAPK-, PI3K-Akt activity was increased when correlated to therapy outcome (**Supplemental Figures 3–7**), thereby verifying the influence of CAF-associated signaling on outcome in EOC.

## 4 Discussion

Among the four EOC subtypes mentioned in the introduction, HGSOC is the most aggressive (4, 7). Fortunately, its high proliferation makes HGSOC rather susceptible towards cytotoxic chemotherapy (3–5, 7). For this reason, chemotherapy has been a cornerstone in clinical HGSOC management for decades (8–10). One of the biggest problems, however, is the disease relapse of HGSOC after initial tumor regression upon receiving chemotherapy (10, 12). This occurs in the majority of patients, creating a crucial necessity to explore the mechanical background behind those relapses and also to provide biomarkers that help stratifying patients for chemotherapy (10, 12).

A detailed look into the activities of the tumor microenvironment (TME) may be helpful to unveil reasons for poor therapy outcome in HGSOCs. As a key factor within TME, CAFs employ miscellaneous functions. One of their best-known functions is the generation of fibronectin and collagen, two substantial components of stroma tissue and the extracellular matrix. Simultaneously, they counterbalance this activity by production of matrix-metalloproteases (25, 28, 33). The amassment of stroma within tissue is also called desmoplasia or DSR, a process often encountered in tumors (51, 52). Besides organization of the extracellular matrix, CAFs employ humoral functions as well by releasing various cytokines. TGF-β is perhaps the most well-studied of them. It has a multitude of different functions like protecting cells from apoptosis and enabling cell cycle arrest. Furthermore, it regulates the immune system by inhibiting effector functions of CD8 positive lymphocytes, NK cells and dendritic cells, while simultaneously promoting regulatory T cells (53–55). All these effects can also benefit the tumor, thereby explaining TGF-βs’ often perceived dual role in cancer (56). While many effects of TGF-β are mediated *via* the SMAD signaling cascade (56, 57), it may also initiate factors related to MAPK and PI3K-Akt signaling pathways (non-canonical TGF-β signaling) (58, 59). These pathways are linked with cell proliferation as well as migration, thereby also enhancing tumor progression (31, 32).

Based on histological and genetic subtyping, five variants of HGSOC can be distinguished. One of them is the mesenchymal subtype which is characterized by occurrence of DSR. Furthermore, this subtype is also associated with a poor survival prognosis (22, 25, 60). Therefore, we aimed to identify DSR in HGSOC patients and link it to poor outcome after platinum-based chemotherapy in patients, defined as having an RFS shorter than 6 months after therapy completion. DSR was supposed to be identified based on the expression of specific genes and activity of specific signaling pathways like MAPK, PI3K-Akt and TGF-β signaling.

Of the six genes associated with therapy failure, CGA and MMP13 are certainly the most outstanding, since they were also linked to reduced OS and RFS, respectively (**Supplemental Figure 1**). MMP13 plays a crucial role for epithelial-mesenchymal transition (EMT) and therefore for cancer progression (61). Furthermore, HIF-1a induced MMP13 expression appears to promote invasion and metastasis in ovarian cancer as well (62). CAFs can also induce EMT *via* secretion of TGF-β1, which then leads to invasion and metastasis (40). The secretion of TGF-β by CAFs additionally promote MMP13 activity (63, 64). CGA encodes for the conserved alpha chain of human gonadotropins (LH, FSH, hCG). In ovarian cancer, the levels of gonadotropins (LH/FSH) are increased. Additionally, it seems they are able to facilitate invasion and metastasis by overexpression of cyclooxygenase-2 (65). Chorionic gonadotropin levels are significantly increased when comparing benign and malignant tumors. Also, gonadotropin levels moderately correlate with tumor staging and grading (66). Taken together, both MMP13 and CGA are known to facilitate invasion and metastasis, which does explain their well-founded impact on patients’ outcome (**Supplemental Figure 1**). MMP13 is also strongly linked to CAFs and TGF-β signaling (40, 63, 64).

EPHA3 is a receptor tyrosine-kinase that is involved in various cell-cell interactions. It is implicated to influence angiogenesis and metastasis among other factors in various malignancies (67), especially gastric cancer (68, 69). PSMD9 is a subunit of the 26s proteasome and is mainly known for regulatory functions. Low expression of PSMD9 was discussed as a biomarker to assess patients’ suitability for radiation therapy in breast cancer, since cells with low expression were more vulnerable for radiation treatment (70). The transcription factor PITX2 has already been investigated in ovarian cancer (71, 72). This factor promotes tumor invasion and is activated by TGF-β and Activin-A (72). Apparently PITX2 is also an important instigator of epithelial-to-mesenchymal transition in ovarian cancer (72). PITX2 and EPH3, especially, are both linked to cancer progression and metastasis, thereby correlating with patients displaying poor therapy outcome. Additionally, PITX2 expression induced by CAFs can initiate EMT (40, 72). It is indicated, that PITX2 activity is enhanced by TGF-β *via* SMAD signaling in patients displaying poor therapy outcome within our cohort (**Figure 5A**).

Though TGF-β signaling, more specifically SMAD-mediated TGF-β signaling, may be considered to be strongly activated according to our results, it should be noted that all downstream components appear weakly expressed, when compared to Cytokine-Cytokine signaling, MAPK signaling or PI3K-Akt signaling (**Figures 4B**, **5B, C**). This led us to the conclusion that TGF-β could function by non-canonical signaling *via* Ras, MAPK and PI3K-Akt. Facilitated by TGF-β, they also promote CAF activity (31, 32, 39, 40, 58, 59). **Supplemental Figure 2** displays a strong expression of FGF, PDGF and HGF in patients with poor therapy outcome. All three factors are strongly linked to CAF activity and DSR (39, 73). An enhanced DSR is also associated with resistance to chemotherapy as drug delivery is compromised by the physical barrier provided by the stroma (40, 74). Summing up, the correlation of MMP13 and PITX2 with poor therapy outcome (**Table 1**) as well the strong gene expression in CAF-associated pathways (**Supplemental Figure 2** and **Figures 4**, **5**) suggest an important role of DSR in patients responding poorly to chemotherapy. CAFs and associated processes have been studied extensively in EOCs (75–77) and even HGSOCs (78). Our study contributes to present knowledge by adding a direct comparison of CAF-associated signaling pathways in both HGSOC and EOC in general. Taken together, our study underlines the prognostic value of CAFs and its importance for clinical decisions. As to this day poor response to platinum-based chemotherapy is a common problem in HGSOC, predictive biomarkers are urgently needed for the development of individualized treatment regimens. Patient stratification for occurrence of DSR or CAFs before platinum-based may be promising for development of models to predict patients’ therapy response in the future.

## Data Availability Statement

The original contributions presented in the study are included in the article/**Supplementary Material**. Further inquiries can be directed to the corresponding author.

## Ethics Statement

The studies involving human participants were reviewed and approved by Ethics Committee of the Medical Faculty of the University Duisburg-Essen (protocol no. 16-6916-BO). The patients/participants provided their written informed consent to participate in this study.

## Author Contributions

Conceptualization: PB and FM. Methodology: MW, EM, SB, and FM. Software: MW and FM. Validation: EM, SB, AB, PM, PB, and FM. Formal analysis: MW, EM, SB, AB, PM, PB, and FM. Investigation: MW, EM, SB, AB, and PB. Resources: KS and RK. Data curation: MW, EM, SB, AB, PM, PB, and FM. Writing-original Draft preparation: MW, PB, and FM. Writing-review and editing: All authors. Visualisation: MW and FM. Supervision: FM. Project administration: KS, RK, PB, and FM. Funding acquisition: KS, RK, PB, and FM. All authors have read and agreed to the published version of the manuscript.

## Conflict of Interest

The authors declare that the research was conducted in the absence of any commercial or financial relationships that could be construed as a potential conflict of interest.

## Publisher’s Note

All claims expressed in this article are solely those of the authors and do not necessarily represent those of their affiliated organizations, or those of the publisher, the editors and the reviewers. Any product that may be evaluated in this article, or claim that may be made by its manufacturer, is not guaranteed or endorsed by the publisher.
