# Supplementary material for: CAF-Associated Paracrine Signaling Worsens Outcome and Potentially Contributes to Chemoresistance in Epithelial Ovarian Cancer
Source: Front Oncol. 2022 Mar 3;12:798680. doi: 10.3389/fonc.2022.798680 (PMC8927667; doi:10.3389/fonc.2022.798680)
Supplement: Supplementary file 1 [file DataSheet_1.docx]

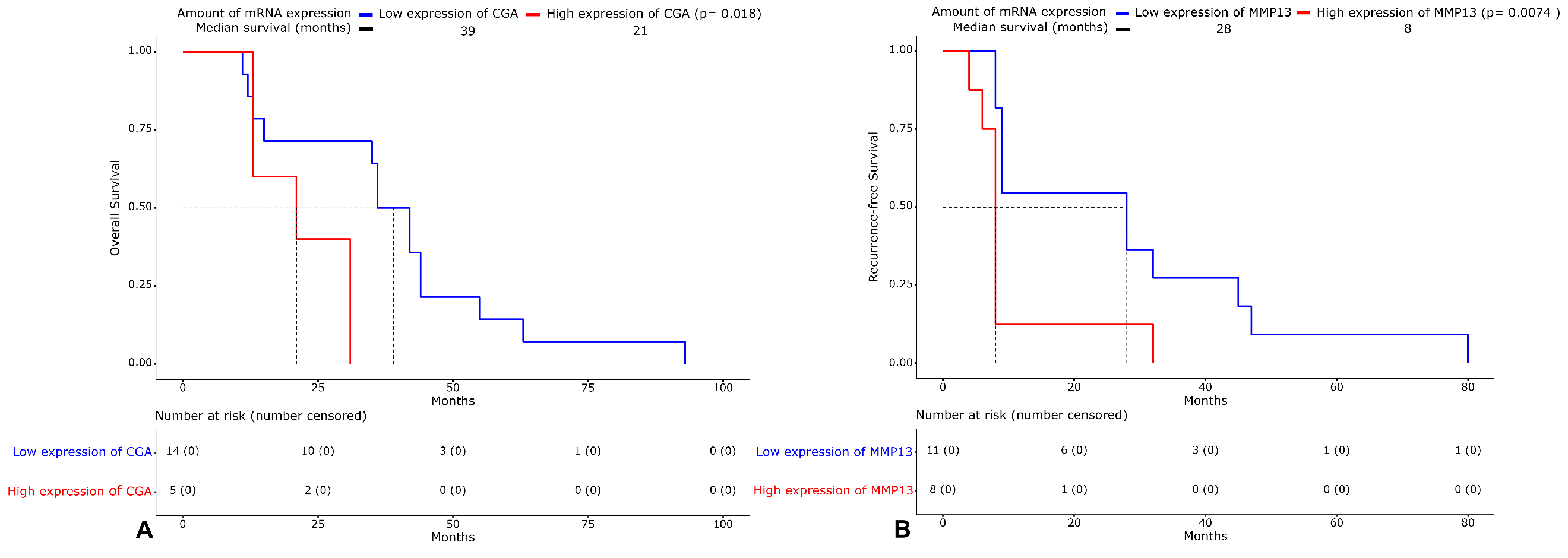


**Supplemental figure 1:** Survival estimates of patients expressing CGA **(A, Overall survival)** and MMP13 **(B, Recurrence-free survival)**. Survival rates were calculated using Cox proportional hazard models and patients (n=19) were separated into two different groups, whether both genes were highly or low expressed. Of the original 24 patients available, only 19 were used in the calculations. Five patients were excluded due to missing survival data. The p-value was outputted by Score-logrank test.


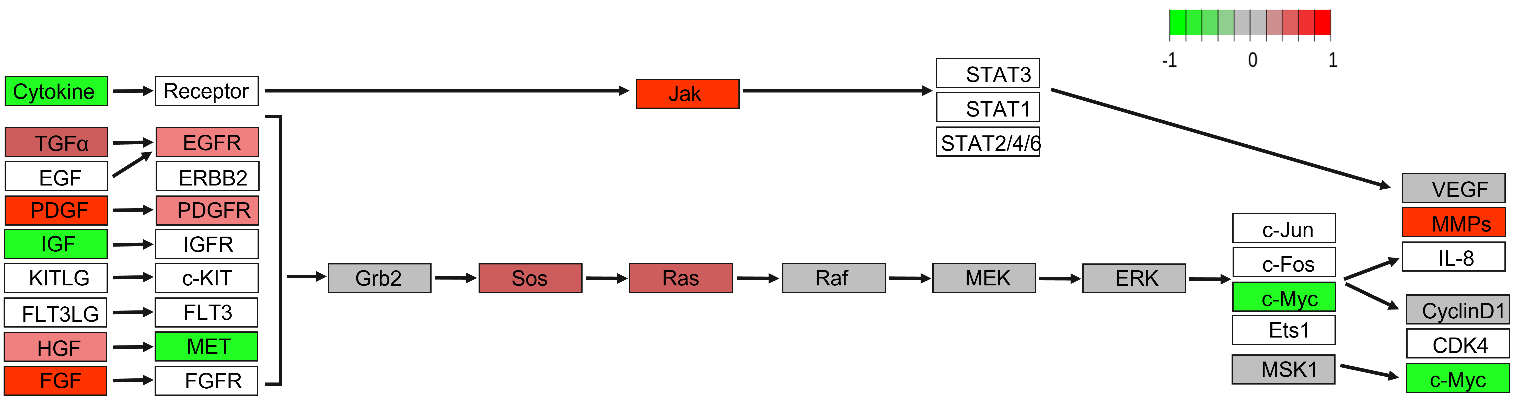


**Supplemental figure 2:** Genes expressed in association with “Pathways in Cancer” (Small excerpt only containing the “Cytokine-cytokine receptor interaction”) and therapy outcome in HGSOC patients. The color code indicates at differential gene expression whether the patients did (green) or did not respond well to chemotherapy (red). This original molecular network map stems from the Kyoto Encyclopedia of Genes and Genomes (KEGG) database.


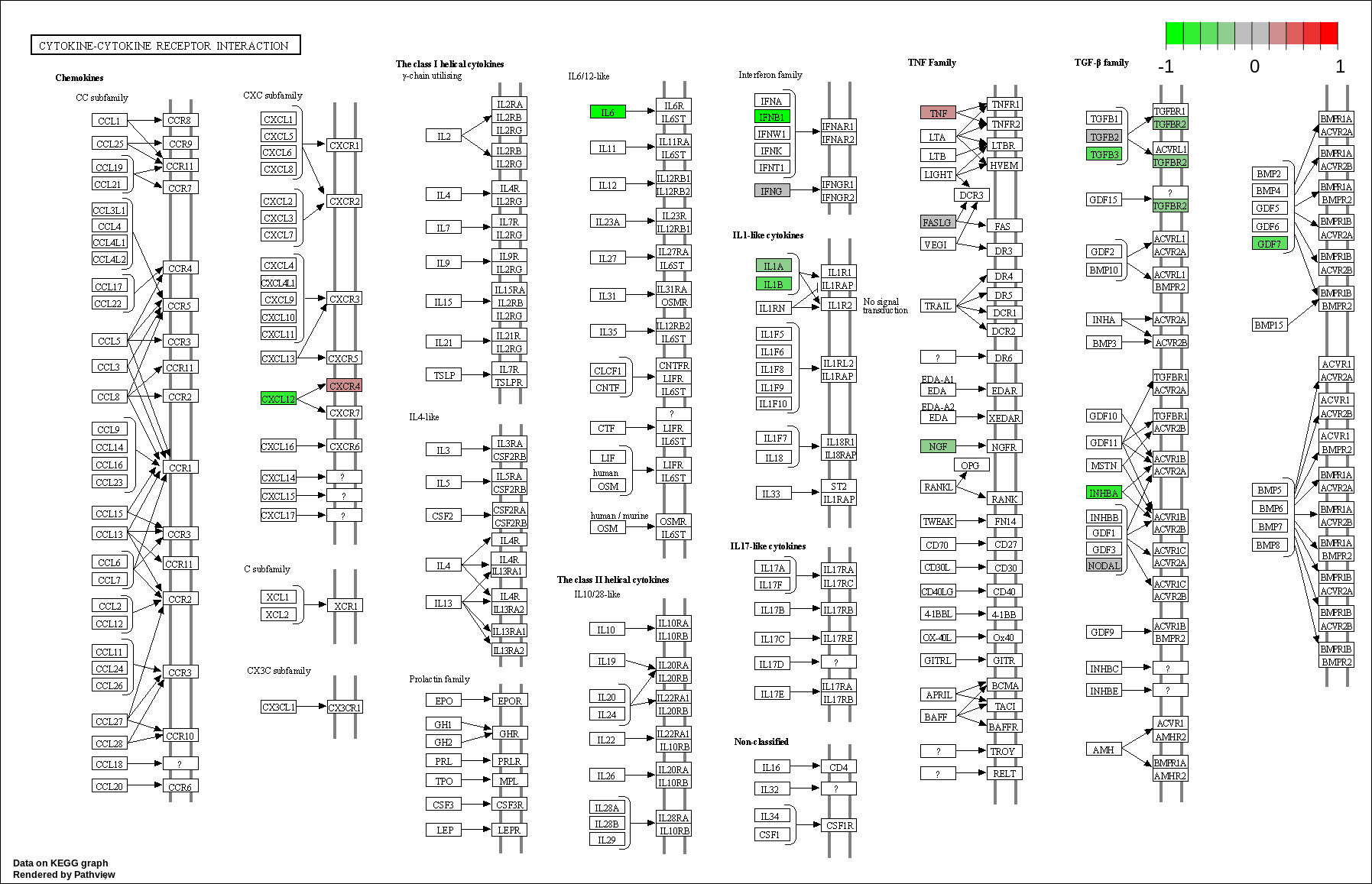


**Supplemental figure 3:** Genes expressed in association with “Cytokine-cytokine receptor interaction” and therapy outcome in the heterogenous epithelial ovarian cancer validation cohort. The color code indicates at differential gene expression whether the patients did (red) or did not respond well to chemotherapy (green). This molecular network map stems from the Kyoto Encyclopedia of Genes and Genomes (KEGG) database.


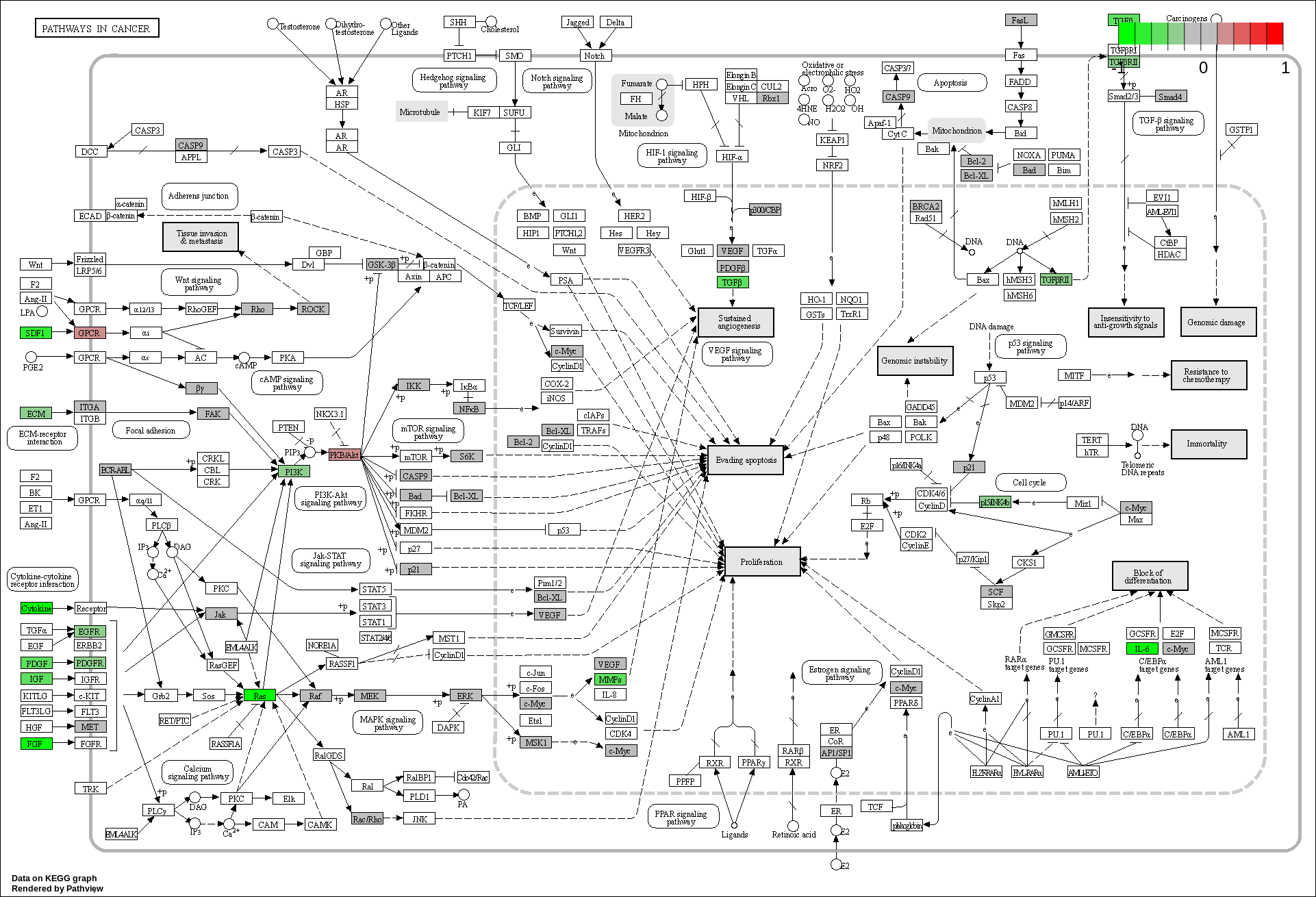


**Supplemental figure 4:** Genes expressed in association with various cancer-related signaling pathways and therapy outcome in the heterogenous epithelial ovarian cancer validation cohort. The color code indicates at differential gene expression whether the patients did (red) or did not respond well to chemotherapy (green). This molecular network map stems from the Kyoto Encyclopedia of Genes and Genomes (KEGG) database.


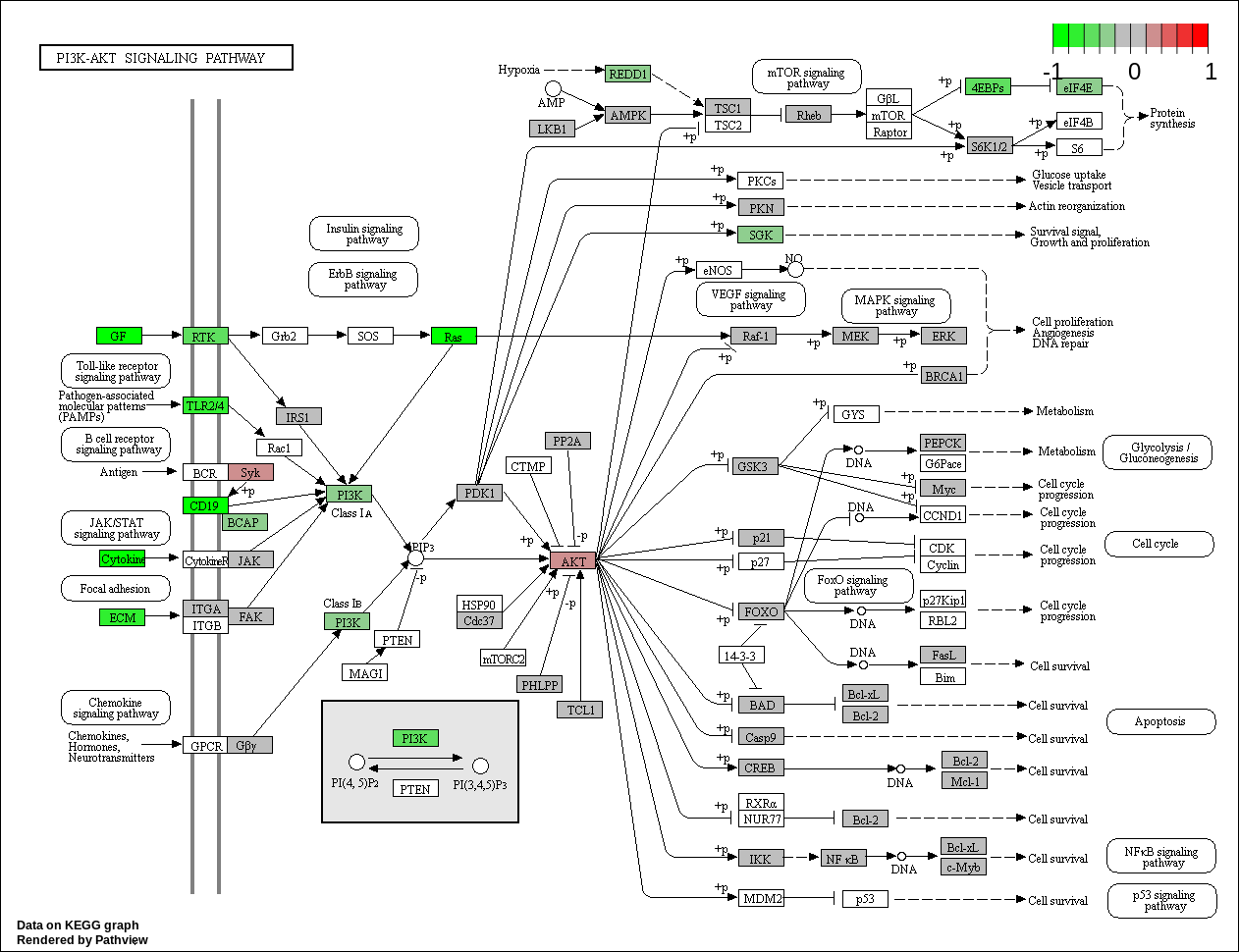


**Supplemental figure 5:** Genes expressed in association with the PI3K-AKT signaling pathway and therapy outcome in the heterogenous epithelial ovarian cancer validation cohort. The color code indicates at differential gene expression whether the patients did (red) or did not respond well to chemotherapy (green). This molecular network map stems from the Kyoto Encyclopedia of Genes and Genomes (KEGG) database.


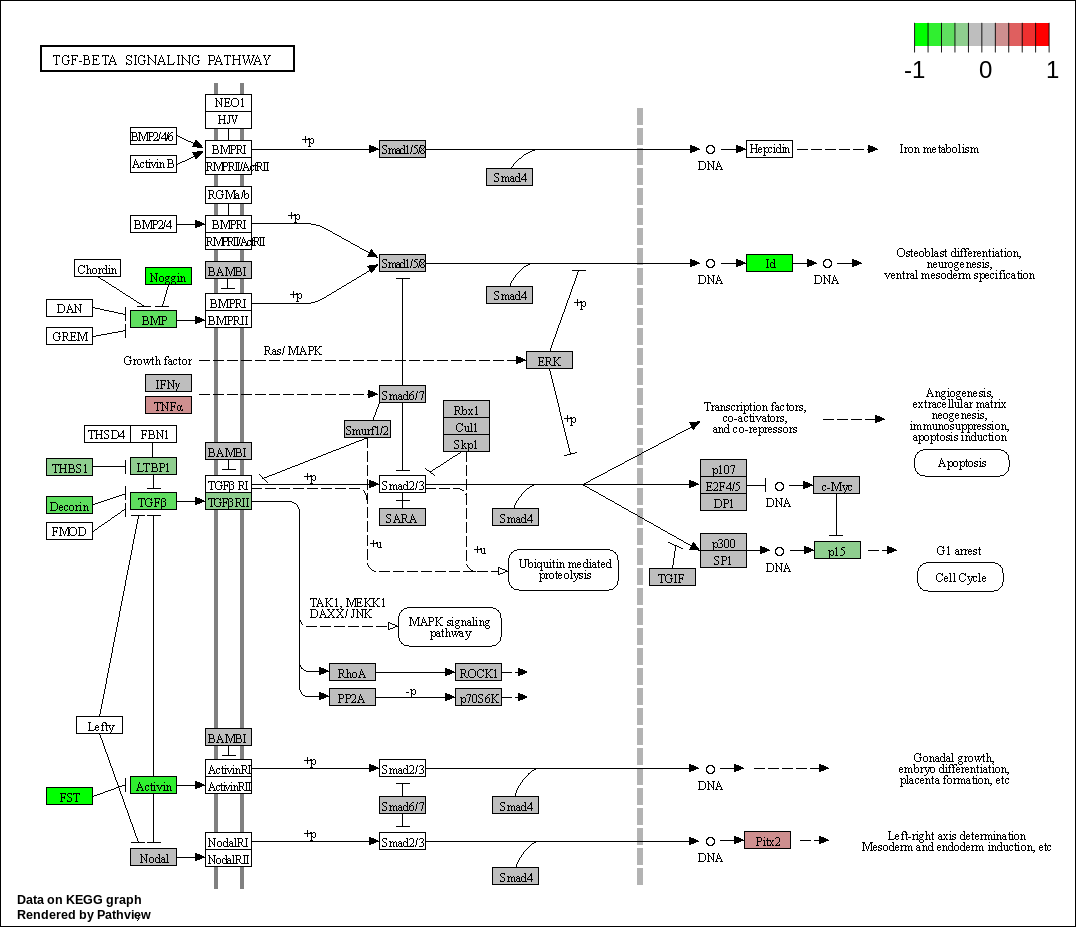


**Supplemental figure 6:** Genes expressed in association with the TGF-ẞ signaling pathway and therapy outcome in the heterogenous epithelial ovarian cancer validation cohort. The color code indicates at differential gene expression whether the patients did (red) or did not respond well to chemotherapy (green). This molecular network map stems from the Kyoto Encyclopedia of Genes and Genomes (KEGG) database.


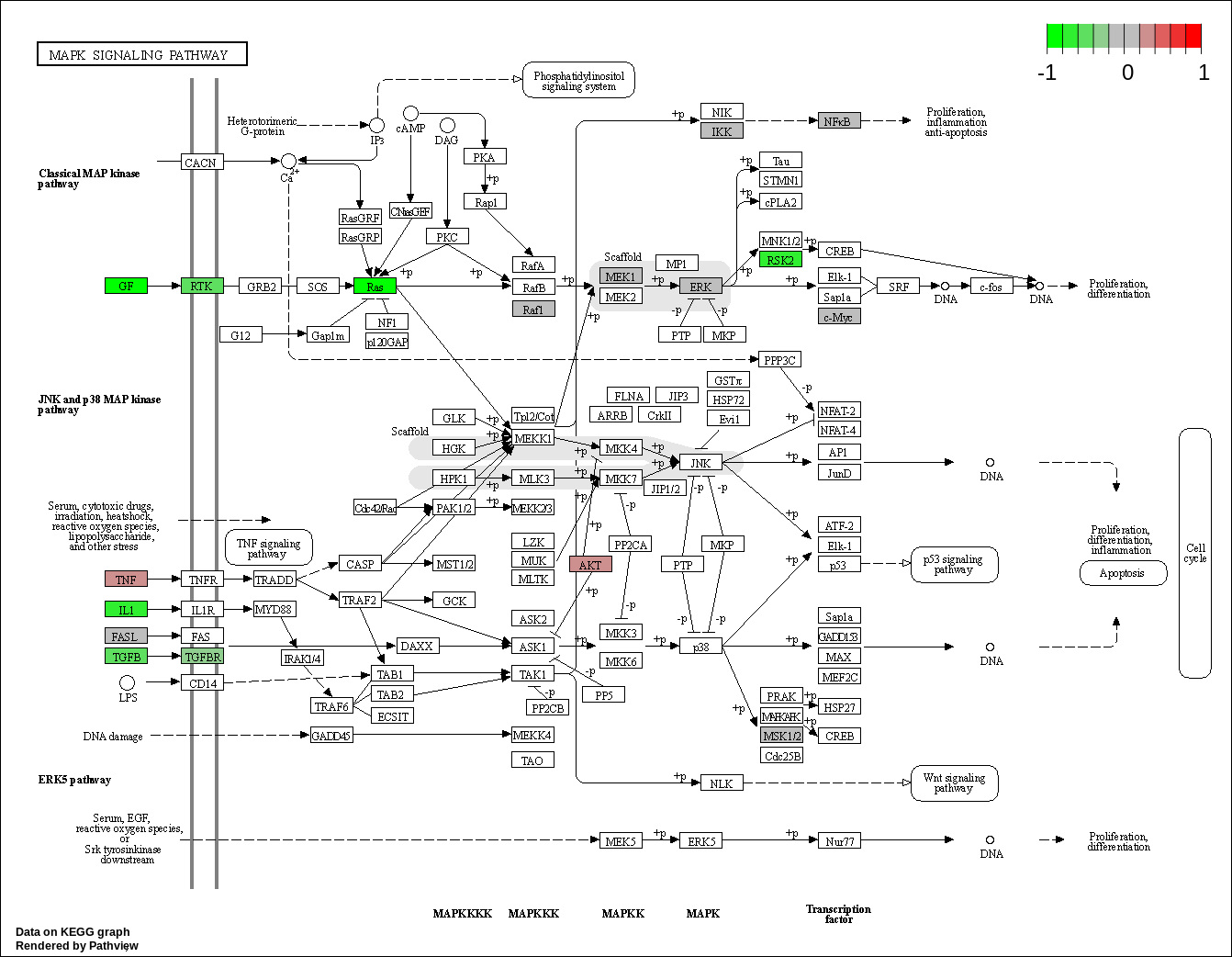


**Supplemental figure 7:** Genes expressed in association with the MAPK signaling pathway and therapy outcome in the heterogenous epithelial ovarian cancer validation cohort. The color code indicates at differential gene expression whether the patients did (red) or did not respond well to chemotherapy (green). This molecular network map stems from the Kyoto Encyclopedia of Genes and Genomes (KEGG) database.
